# Exploring the association between cognitive decline and all-cause mortality with blood pressure as a potential modifier in oldest old individuals

**DOI:** 10.1038/s41598-022-21487-8

**Published:** 2022-10-12

**Authors:** Jun Duan, Napoleon Bellua Sam, Shi-Jia Wang, Yan Liu

**Affiliations:** 1 Department of Medical Record Statistics, Peking University Shenzhen Hospital, No. 1120, Lianhua Road, Futian District, Shenzhen, 518036 China; 2 Department of Medical Research and Innovation, School of Medicine, University for Development Studies, Tamale, N/R Ghana; 3 Department of Cardiovascular Medicine, Peking University Shenzhen Hospital, Shenzhen, China

**Keywords:** Hypertension, Epidemiology, Risk factors, Neurological disorders

## Abstract

Few studies have systematically explored the association between cognitive decline and all-cause mortality among oldest old individuals (above 80 years old), and there is limited evidence of blood pressure (BP) as a potential effect modifier. Therefore, this study included 14,891 oldest old individuals (mean age: 90.3 ± 7.5 years); 10,904 deaths and 34,486 person-years were observed. Cognitive scores were calculated using the Chinese version of the Mini-Mental State Examination (MMSE). Cognitive decline was stratified into ten categories (C0–C9). Continuous cognitive scores were used to assess the interactions of modifiers of the cognitive decline and all-cause mortality association and potentially modifiable factors. Potential effect modifiers were explored by age, sex, BP status and hypertension. Cox proportional hazards models were used to evaluate the relationship between cognitive decline and all-cause mortality after adjustments for demographic characteristics, socioeconomic status, lifestyle factors, leisure activities and health conditions. Participants who progressed to severe cognitive impairment from high normal cognitive function (C3), low normal cognitive function (C6), or mild cognitive impairment (C8) had 55%, 56%, and 63% higher mortality risks, respectively, than those who maintained high normal cognitive function (C0). The multivariate-adjusted model indicated that oldest old individuals with a decrease of more than one point in the MMSE score per year had an approximately 4% all-cause mortality risk. The relationship between cognitive decline and mortality was statistically influenced by sex (P = 0.013), high BP in nonagenarians (P = 0.003), and hypertension (P = 0.004) but not by age (P = 0.277). Our findings suggest that periodic screening for cognitive decline and strengthening BP management may be necessary for public health.

## Introduction

Cognitive decline, a clinical intermediate state between normal cognitive aging and dementia, is a major and increasing health problem worldwide^1–3^. Previous prospective studies largely focused on the cognitive impairment-mortality relationship, but reports of the association of cognitive decline with mortality are scarce^4–7^, and many potential effect modifiers of this relationship are still unclear.

Currently available evidence indicates that the results are inconsistent, which may be due to differences in sample sizes, variations in analyses, and differences in respondent characteristics, including age, sex, and ethnicity. In both 9-year (mean age = 77.6) and 6-year (mean age = 82.5) longitudinal studies, 3-year interval cognitive decline measured by the Mini-Mental State Examination (MMSE) was related to all-cause mortality in the subsequent 6-year or 3-year period^8,9^. However, the large confidence intervals indicated inadequate power or a small sample size, and the association between cognitive decline and mortality might not have been thoroughly assessed considering the different stages of cognitive decline due to inaccurate stratification of cognitive decline by the median.

Other significant factors in addition to age and sex, including blood pressure (BP) and the consequences of high BP, such as hypertension, might also affect the relationship between cognitive decline and mortality, yet few studies have reported^10,11^. In particular, some studies have shown that the risk of mortality associated with cognitive impairment and with cognitive decline decreases with age^8,9,12^; this might seem counterintuitive and might mislead the public because little is known about how age modifies the cognitive decline–mortality risk, especially in oldest old individuals (aged 80 and older). In addition, high BP might lead to an increase in disability-adjusted life years, which could potentially affect more than 75% of persons over 75 years worldwide^13^. High BP has also been identified as a risk factor for cognitive impairment and dementia^14^. We therefore formulated the hypothesis that high BP would have a modification effect on the association between cognitive decline and mortality in oldest old individuals.

The main objective of this study was to evaluate the relationship between cognitive decline, stratified by level, and all-cause mortality in oldest old individuals based on a large prospective cohort study in China and to examine how BP and hypertension, in addition to age and sex, modify the relationship between cognitive decline and all-cause mortality.

## Methods

### Study design and participants

The Chinese Longitudinal Healthy Longevity Survey (CLHLS) is a nationwide survey with the largest sample of participants aged 80 years and older in the world.

Until recently, the program randomly selected half of the cities and counties in 23 provinces of China. The 20-year prospective cohort study began in 1998, with subsequent follow-up and recruitment of new participants in 2000, 2002, 2005, 2008, 2011, 2014, and the participants were followed until 2018. A more detailed description of the CLHLS has been published elsewhere^15^. The study design of the CLHLS and the enrollment of participants are described in Supplementary Materials 1 and 2, respectively.

### Definitions of cognitive decline and all-cause mortality

Cognitive decline was evaluated using the Chinese version of the MMSE, a widely used cognitive test^16,17^. Cognitive decline was defined as a decrease in the MMSE score from baseline at 2- to 3-year intervals^8^. First, baseline MMSE scores were divided into four categories: severe cognitive impairment (0–17), mild cognitive impairment (18–23), low normal cognitive function (24–27) and high normal cognitive function (28–30) (Supplementary Material 3). Second, cognitive decline was defined as a decrease in the MMSE score between from the first cognitive function test to the second cognitive function test, which was administered at a 2- to 3-year interval. For example, high normal cognitive function was considered to have deteriorated to low normal cognitive function, mild cognitive impairment, or severe cognitive impairment if the MMSE scores decreased by 1–6 points, 5–12 points and 11–30 points, respectively. There are six main forms of cognitive decline that occur at various stages. MMSE scores that remained the same or increased at two different time periods were representative of cognitive function maintenance. Therefore, there are four ways to maintain cognitive function. In Supplementary Material 3, the divisions are described in further detail. The rate of change in the MMSE score, which was calculated as the difference between the baseline cognitive function test score and the second cognitive function test score divided by the follow-up time ((MMSE score at baseline—MMSE score at the second cognitive function test)/the interval between the two follow-ups in years), was used to define cognitive decline and was considered a continuous variable. All-cause mortality that occurred between 1998 and 2018 during the follow-up survey was the primary outcome. To determine if respondents completed the study, died (with the date of death), or were lost to follow-up, an elderly subject’s survival status was obtained from family members or relatives during the follow-up survey in 2018. The researchers did attempt to locate or contact elderly individuals who were lost to follow-up. Censored data included the data of elderly individuals who survived but were unable to be contacted after follow-up.

### Definitions of covariates

A standardized questionnaire was designed to collect data on the following variables: demographic characteristics, socioeconomic status, lifestyle factors, leisure activities and health conditions. (1) Demographic characteristics included sex (male or female) and age (as a continuous variable). (2) Socioeconomic status included residence (urban or rural), educational background (illiterate or not illiterate), current spouse status (have spouse or have no spouse), and living pattern (with family members or not). (3) Lifestyle factors included regular exercise (yes/no), current smoking (yes/no), current alcohol consumption (yes/no), and dietary diversity (DD) (yes/no). (4) Leisure activities were divided into 3 response categories (never, sometimes, and often) and included doing housework, reading, watching TV or listening to the radio, keeping pets and gardening. (5) Health conditions included high BP (systolic BP (SBP) > 140 mmHg or diastolic BP (DBP) > 90 mmHg, yes or no), disability in activities of daily living (ADL, yes/no), hypertension (yes or no), heart disease (yes or no), and respiratory disease (yes or no). Covariates were assessed at baseline. In addition, educational background was categorized as “not illiterate” if the participant had received > 1 year of any formal education and “illiterate” if the participant had not received any formal education. Current spouse status referred to whether the current spouse was alive or not. Regular exercise referred to purposeful fitness activities, such as walking, playing ball, running, etc. DD was assessed according to the consumption of nine major food groups (meat, fish and seafood, eggs, beans, fruits, vegetables, tea, garlic, and sugar or candy) and defined as poor if the DD value was lower than the mean value (3.04)^18^. Six activities were considered ADL, including dressing, bathing, using the toilet, continence, getting in/out of bed or a chair and eating. ADL impairment was defined as an inability to perform any one of the tasks independently^19^. SBP/DBP measurements were conducted using a mercury sphygmomanometer by trained internists. Cardiovascular and respiratory disease histories were collected by self-reported questions.

### Statistical analysis

Missing data accounted for less than 1.1% of covariates, and mean value imputation methods were applied to account for the missing covariate values. Means and standard deviations were summarized for continuous variables, and frequencies and percentages were summarized for categorical variables. Comparisons between elderly groups were conducted using the Cochran–Mantel–Haenszel test for categorical variables and the Kruskal‒Wallis test for continuous variables. This study calculated hazard ratios (HRs) and 95% confidence intervals (CIs) using Cox proportional hazards models. Stepwise regression was used to determine the independent risk factors for mortality and the important confounders identified in previous studies. Several models were developed: model 1 was adjusted for demographic characteristics; model 2 was adjusted for the variables in model 1 plus socioeconomic status and lifestyle factors; model 3 was adjusted for the variables in model 2 plus leisure activities; and model 4 was adjusted for the variables in model 3 plus health conditions. To avoid collinearity, model 4 did not include hypertension. Maintenance of high normal cognitive function was considered the reference. Kaplan‒Meier analysis was used to construct survival curves according to cognitive status, and the survival curves were compared by the log-rank test. This study tested the suitability of the proportional risk assumption using hypothesis tests based on Schoenfeld residuals, and the proportional hazards assumption was not severely violated (Schoenfeld P = 0.08, Supplementary Material 4). The follow-up time in years was used as the time interval since enrollment.

Continuous cognitive scores were used to assess the interactions of modifiers of the cognitive decline and all-cause mortality association and potentially modifiable factors. Potential effect modifiers were explored by age, sex, BP and hypertension. Age and BP were the key risk factors and the main targets in the association between cognitive decline and all-cause mortality, so subanalyses stratified by age-at-enrollment and BP (high BP, age 80–89 years (octogenarians); high BP, age ≥ 90 years (nonagenarians); non-high BP, 80–89 years; non-high BP, age ≥ 90 years) were performed. In addition, to differentiate this study from previous studies, we included a younger age group (age range 65–79) to verify our hypothesis, as presented in the appendix materials.

For further analysis, we employed the following analytical approaches to check the robustness of the primary results: (1) we excluded aged individuals whose MMSE scores increased; (2) we excluded individuals with comorbidities (hypertension, heart disease or respiratory disease); and (3) we excluded those who died in the first 0.5, 1 and 1.5 years due to the possibility that the decrease in cognitive performance before mortality and/or disease progression in the last year of life might influence the results. (4) Additionally, to evaluate whether the associations differed according to different follow-up times and reverse causation, we stratified across time strata by the median (3 years) follow-up period.

Data analysis was conducted using R version 3.3.4 with the “survival” package. All statistical tests were two-tailed, and statistical significance was judged by *P*-values < 0.05.

### Ethics approval and consent to participate

The Protection of Human Subjects for the CLHLS was approved by the biomedical ethics committee of Peking University (IRB00001052-13074). The study was performed in accordance with relevant guidelines and regulations. Written informed consent was obtained from all participants and/or their relatives.

## Results

### Baseline characteristics

Supplementary Material 5 presents the detailed baseline characteristics of the study participants in six successive and nonoverlapping cohorts classified by cognitive decline level. A total of 14,791 oldest old individuals were included, and the mean age was 90.3 years. A total of 10,904 deaths and 34,486 persons-years were observed in the 20-year prospective cohort study. Those with various levels of cognitive decline tended to be older, with ages ranging from 86.4 to 91.3, 87.9 to 92.9, and 90.1 to 93.8 years, than those who retained cognitive function. As cognitive decline progressed, participants were more likely to be female, be illiterate, live in an urban area, have no spouse, live without family members, not engage in regular exercise, be nonsmokers, be nondrinkers, have poor DD, have ADL impairment, not engage in regular leisure activities, and have no heart disease or high BP.

### Association between cognitive decline and mortality

Table 1 shows that the risk of mortality increased in parallel with cognitive decline in all ten cognitive decline categories (C0–C9). After adjustments for demographic characteristics, socioeconomic status, lifestyle factors, leisure activities, and health status, cognitive function progressed to severe cognitive impairment in those with high normal cognitive function, low normal cognitive function, and mild cognitive impairment initially, with HRs of 1.55 (95% CI 1.42, 1.69), 1.56 (95% CI 1.42, 1.72), and 1.63 (95% CI 1.47, 1.80), respectively, compared to those who maintained high normal cognitive function. High normal cognitive function and low normal cognitive function were associated with HRs of 1.25 (95% CI 1.14, 1.38) and 1.17 (95% CI 1.05, 1.30), respectively, when cognitive function declined to mild cognitive impairment.

**Table 1:** Association of cognitive decline with mortality after adjustment for different covariates.

MMSE score	Hazard ratio (95% CI)			
	Model 1	Model 2	Model 3	Model 4
Baseline high normal cognitive function				
High normal, maintain function	1.00 (Reference)	1.00 (Reference)	1.00 (Reference)	1.00 (Reference)
High normal to low normal	1.13 (1.04, 1.22)	1.12 (1.04, 1.21)	1.10 (1.02, 1.19)	1.10 (1.02, 1.19)
High normal to mild impairment	1.30 (1.18, 1.42)	1.28 (1.17, 1.40)	1.26 (1.15, 1.38)	1.25 (1.14, 1.38)
**High normal to severe impairment**	**1.61 (1.47, 1.75)**	**1.59 (1.46, 1.73)**	**1.54 (1.41, 1.68)**	**1.55 (1.42, 1.69)**
Low normal, maintain function	1.16 (1.08, 1.25)	1.15 (1.06, 1.23)	1.09 (1.01, 1.18)	1.07 (0.99, 1.15)
Low normal to mild impairment	1.26 (1.14, 1.40)	1.25 (1.13, 1.39)	1.19 (1.08, 1.32)	1.17 (1.05, 1.30)
**Low normal to severe impairment**	**1.75 (1.60, 1.92)**	**1.72 (1.57, 1.89)**	**1.60 (1.46, 1.76)**	**1.56 (1.42, 1.72)**
Mild impairment, maintain function	1.25 (1.16, 1.35)	1.22 (1.13, 1.32)	1.15 (1.06, 1.24)	1.10 (1.00, 1.20)
**Mild impairment to severe impairment**	**1.94 (1.78, 2.11)**	**1.89 (1.73, 2.07)**	**1.72 (1.57, 1.88)**	**1.63 (1.47, 1.80)**
Severe impairment	1.71 (1.60, 1.83)	1.66 (1.55, 1.78)	1.50 (1.40, 1.61)	1.32 (1.15, 1.53)

Model 1: adjusted for demographic characteristics (sex and age); Model 2: adjusted for the covariates in model 1 plus socioeconomic status (residence, educational background, current spouse status, marital status and living pattern) and lifestyle factors (regular exercise, current smoking status, current drink status, dietary diversity (DD)); Model 3: adjusted for the covariates in model 2 plus leisure activities (housework, reading, watching TV and listening to the radio, keeping pets and growing flowers); Model 4: adjusted for the covariates in model 3 plus health conditions (high blood pressure (BP), disability in activities of daily living (ADL), and respiratory disease). Significant values are in bold.

### Potential effect modifiers of the cognitive decline–mortality association

**Figure 1:**
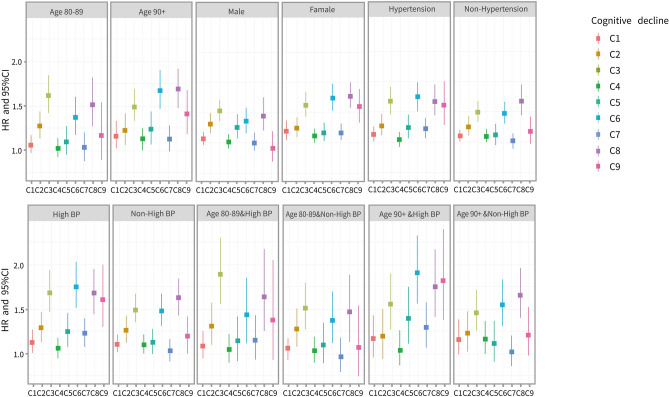
Potential effect modifiers of the association of cognitive decline, stratified into ten categories, with mortality after full adjustment for covariates. Adjustments for demographic characteristics (sex and age), socioeconomic status (residence, educational background, current spouse status, and living pattern), lifestyle factors (regular exercise, current smoking, current alcohol consumption, dietary diversity (DD)), leisure activities (housework, reading, watching TV or listening to the radio, keeping a pet and gardening) and health conditions (high blood pressure (BP), disability in activities of daily living (ADL), hypertension, and respiratory disease). Cognitive decline: Ten categories (C0–C9, compared to C0): *C0* High normal cognitive function maintenance, *C1* High normal cognitive function decline to low normal cognitive function, *C2* High normal cognitive function decline to mild cognitive impairment, *C3* High normal cognitive function decline to severe cognitive impairment, *C4* Low normal cognitive function, maintain function, *C5* Low normal cognitive function decline to mild cognitive impairment, *C6* Low normal cognitive function decline to severe cognitive impairment, *C7* Mild cognitive impairment maintenance, *C8* Mild cognitive impairment maintenance, *C9* Severe cognitive impairment maintenance.

Figure 1 illustrates the association between detailed cognitive decline and mortality, estimated in relation to modified variables (age, sex, high BP, and hypertension). The risk of all-cause mortality was higher in nonagenarians with baseline MMSE scores that decreased to severe impairment levels from low normal (C6) and mild cognitive impairment (C8) levels, with HRs of 1.67 (95% CI 1.47, 1.89) and 1.68 (95% CI 1.46, 1.92), respectively, than in their octogenarian counterparts, with HRs of 1.37 (95% CI 1.16, 1.61) for C6 and 1.51 (95% CI 1.26, 1.82) for C8 cognitive impairment. Patients with cognitive decline with high BP had a substantially higher risk of mortality, with HRs of 1.67 (95% CI 1.45, 1.93), 1.74 (95% CI 1.50, 2.02) and 1.67 (95% CI 1.42, 1.96), than patients with cognitive decline without high BP, with HRs of 1.49 (95% CI 1.33, 1.67), 1.46 (95% CI 1.30, 1.67) and 1.62 (95% CI 1.41, 1.84). The effect on the cognitive decline–mortality association was consistent in subanalyses that stratified the data by age and BP condition. More detailed results are described in Fig. 1.

When cognitive decline was considered as a continuous variable, the mortality risk was 1.04 (95% CI 1.04, 1.05). The *P* values for the interactive effects of age, sex, high BP and hypertension on the continuous cognitive decline–mortality association were 0.277, 0.013, 0.082 and 0.004, respectively. After age stratification, the *P* values for the interactions were 0.173 among 80- to 89-year-olds and 0.002 among 90-year-olds and older (Table 2).

**Table 2:** Interaction effects on the association of cognitive decline, stratified as continuous categories, with mortality after adjustments for corresponding covariates.

Subgroup	No. of patients	HR (95% CI)	HR (95% CI) for interaction	P for interaction
Total	14,891	1.04 (1.04, 1.05)		
**Age**			0.99 (0.99, 1.00)	0.277
Aged 80–89	7220	1.05 (1.04, 1.06)		
Aged 90+	7671	1.04 (1.03, 1.05)		
**Sex**			1.01 (1.00, 1.01)	0.013
Male	6058	1.04 (1.03, 1.04)		
Female	8833	1.05 (1.04, 1.05)		
**High BP**			1.00 (0.99, 1.02)	0.082
Yes	8589	1.05 (1.04, 1.06)		
No	6259	1.04 (1.03, 1.05)		
**Hypertension**			1.01 (1.00, 1.01)	0.004
Yes	2224	1.06 (1.05, 1.08)		
No	12,667	1.04 (1.03, 1.05)		
**Aged 80**–**89**			1.01 (0.99, 1.02)	0.173
Non-high BP	4049	1.04 (1.03, 1.05)		
High BP	3171	1.06 (1.04, 1.07)		
**Aged 90+**			1.00 (1.00, 1.01)	0.002
Non-high BP	4547	1.04 (1.03, 1.05)		
High BP	3124	1.04 (1.03, 1.05)		

In addition, when we included the younger age group (aged 65–79), significant differences were observed among age groups (*P* < 0.05). Additional details are described in Supplementary Materials 6 and 7.

### Sensitivity analysis

After excluding those with increased cognitive scores, the data still indicated a robust relationship between cognitive decline and mortality (Supplementary Material 8). There was almost no change in the association when those with corresponding diseases (Supplementary Material 9) and mortality that occurred in the first 0.5, 1 and 1.5 years were excluded (Supplementary Material 10). When the analyses were stratified by follow-up time, a higher risk of mortality associated with cognitive decline was observed in the continuous ten cognitive decline categories in those with a follow-up time of more than 3 years, which was in the opposite direction of reverse causation (Supplementary Material 11).

## Discussion

In this national representative prospective cohort study including 14,892 aged individuals, we found that cognitive decline was associated with an elevated risk of all-cause mortality, even in those with a low level of cognitive decline. In addition, our results indicated that female sex, low BP, and the absence of hypertension positively affected the cognitive decline–mortality association.

We have provided new evidence suggesting that even a low degree of cognitive decline increases the risk of all-cause mortality. In addition, this is the first study, to our knowledge, to explore this association among oldest old individuals, stratified by octogenarians and nonagenarians, as well as BP and a high-BP-related condition. The research described cognitive decline and explored the association between cognitive decline and mortality. However, the large CIs indicated inadequate power, which might be due to the small sample size (n = 322)^8^. Recent research also found that cognitive decline measured by the MMSE was related to mortality in the subsequent 3-year period. However, this research may be limited by stratification of cognitive decline according to the median in the assessment of the cognitive decline–mortality association^9^. Both of these studies and others showed that the risk of mortality associated with cognitive decline decreased with age when the population was stratified into those aged 65–79 years and older than 79 years. However, because they did not further stratify those older than 79 years, their results might mislead the public. We also stratified the population using the methods in previous research and obtained consistent results (Supplementary Material 7). Therefore, this study adds new evidence that the risk of mortality associated with cognitive decline increases with age among oldest old individuals.

Our findings also suggest a less-considered mechanism by which cognitive decline potentially influences all-cause mortality. The mechanisms driving the cognitive decline–mortality association with age remain unclear. A previously reported reverse association with age indicated that the cognitive decline–mortality association might be linked more closely to underlying diseases that increase mortality risk (e.g., Alzheimer's disease or cardiovascular disease)^20,21^. This study might not refute this hypothesis even though octogenarians whose baseline cognitive status progressed from low normal or mild cognitive impairment to severe impairment, according to MMSE scores, had a higher mortality risk than nonagenarians, and the trend was the same in both groups. This study highlights the possibility that cognitive decline might have been a contributing factor to mortality among oldest old individuals over the 20-year follow-up period or that it might be a sign of biological aging. In light of the findings of this study, a rapid decline in cognitive function might be a warning sign that indicates the occurrence of specific diseases and the impending end of life.

Additionally, this study found that females were more affected by the association between cognitive decline and mortality than males. A possible explanation is that females whose baseline MMSE score decreased from a high normal cognitive function, low normal cognitive function, or mild cognitive impairment value to a severe cognitive impairment value had a higher mortality risk than males in all ten categories of continuous cognitive decline; this might be attributed to less physical activity and the occurrence of emotional disorders in females^22,23^. However, regarding sex, the results of this study were not consistent with those of previous studies. Males had more traditional lifestyle risk factors than females, such as smoking, alcohol consumption, and physical inactivity, which contributed to the association of cognitive decline and all-cause mortality^24^. The potential reason is still unclear, and more analogous studies analyzing sex and the cognitive decline–mortality association are needed to evaluate our findings.

Higher mortality risks were observed in both older adults and those with high BP. Recent results of a randomized clinical trial and previous research using the CLHLS dataset reported that high BP might lead to poor cognitive function^10^. However, our main goal was not to explore the etiological chain of BP in the cognitive decline–mortality association but to explore the modification effect of BP on this association. As expected, in elderly individuals with high BP, cognitive decline had a greater impact on the risk of mortality. It is possible that collider stratification bias exists. Nevertheless, our results provide evidence of a cognitive decline–mortality association across age and BP strata, suggesting that high BP predisposes nonagenarians to the adverse health effects of cognitive decline. For further analysis, this study used hypertension for verification of this effect. Interaction effects on the cognitive decline–mortality association were also observed for hypertension. According to previous studies, high BP is a sign of widespread atherosclerosis and artery stiffness^25,26^ and might cause decreased perfusion of the cerebral white matter, which is one of the main risk factors for cognitive decline, ultimately increasing the risk for mortality associated with cognitive decline^27,28^. Therefore, we believe that this study provides data support for future clinical and basic research.

The strength of this study is that it is the largest prospective longitudinal study investigating the associations between different types of cognitive decline and all-cause mortality; it had a large sample size of elderly individuals, included survival analysis of the observed data, explored potential effect modifiers, established and included adjustments for potential risk factors, and included robust sensitivity analysis.

However, several limitations should be noted. First, the MMSE is a relatively simple screening tool for cognitive decline with a ceiling effect, and comprehensive neuropsychological diagnosis data to capture all detailed aspects of cognitive function were not available. Second, because cause-of-death information provided by village doctors was not based on formal medical examinations, the records of causes of death and comorbidities contributing to death were inevitably incomplete. This study might have failed to detect a relationship between cognitive decline and mortality due to other competing causes of death, although the analyses were adjusted for multiple factors of incident disease (including hypertension and respiratory disease) in our sensitivity analysis. Third, this study might benefit from analysis of trajectories of cognition rather than scores based on two measurements. Further research and evaluation are needed in the future. Fourth, BP was measured at a single visit, and missing data on the use of antihypertensive medications may diminish the data accuracy. Finally, this study focused on only the Chinese oldest old population. Although it could have been generalized to include other age groups and ethnic groups, such as Western populations, the corresponding covariates were still redefined and reconsidered due to different socioeconomic environments and population characteristics.

## Conclusions

Cognitive decline, even a low level of cognitive decline, was associated with an elevated risk of all-cause mortality among oldest old individuals. A higher mortality risk was observed even after stratifying data by age and BP, especially in nonagenarians. Thus, it is necessary to periodically screen for cognitive decline and strengthen BP prevention and control interventions for public health.

## Supplementary Information

Supplementary Information.

## Data Availability

The CLHLS is an open cohort dataset. We obtained the data from the English site https://sites.duke.edu/centerforaging/programs/chinese-longitudinal-healthy-longevity-survey-clhls/ and the Chinese site https://opendata.pku.edu.cn/. In addition, data that have been cleaned are available from the authors upon request. Please do not hesitate to contact the corresponding author.
