# Supplementary material for: Exploring the association between cognitive decline and all-cause mortality with blood pressure as a potential modifier in oldest old individuals
Source: Sci Rep. 2022 Oct 12;12:17108. doi: 10.1038/s41598-022-21487-8 (PMC9556626; doi:10.1038/s41598-022-21487-8)
Supplement: Supplementary file 1 — Supplementary Information. [file 41598_2022_21487_MOESM1_ESM.doc]

**Supplementary material 1.** Study design of CLHLS and definition and classification of the confounders.

The CLHLS has the largest sample of centenarians in the world. Project objectives are to shed new light on a better understanding of the determinants of healthy longevity of human beings and to determine which factors, out of a large set of social, behavioral, biological, and environmental risk factors, play an important role in healthy longevity. (https://sites.duke.edu/centerforaging/programs/chinese-longitudinal-healthy-longevity-survey-clhls/). Data quality, including assessments of mortality rate, proxy use, non-response rate, sample attrition, reliability and validity of major health measures, and the rates of logically inconsistent answers, have been conducted extensive evaluations with generally satisfactory results compared to other major aging studies[1].

Until present, the program randomly selected half of the cities and counties in 23 provinces of China. It began in 1998, with subsequent follow-up and recruitment of new participants in 2000, 2002, 2005, 2008, 2011, 2014 and followed up until 2018, using the instruments of the Danish longevity survey analyzed by Christensen and colleagues. The instruments were adapted to the Chinese culture and socioeconomic context. Since the 2002 wave, the CLHLS was expanded from only recruiting oldest-old in 1998 and 2000 waves to also interviewing approximately three randomly selected nearby elders aged 65-79 of predefined age and sex in conjunction with every two centenarians. The CLHLS adopted a targeted random-sample design to ensure representativeness, even distribution across age and gender and sufficient sub-sample size of the oldest-old aged 80+, plus compatible young-old aged 65-79.

1. Zeng Y, Feng Q, Hesketh T, Christensen K, Vaupel JW: **Survival, disabilities in activities of daily living, and physical and cognitive functioning among the oldest-old in China: a cohort study**. *LANCET* 2017, **389**(10079):1619-1629.

**Supplementary material 2.** On account of assessed around 2-3 years of cognitive decline on following mortality, our research covered the six successive and non-overlapping cohorts from 1998, 2000, 2002, 2005, 2008 and 2011, followed up until 2018. A total of 43,487 older adults were included at baseline and cognitive function was tested twice. We excluded 9,202 participants aged 79 and younger, 4,968 participants lost-to-follow-up at the first follow-up survey, 13,637 oldest old died at 2-3 years interval and 791 older adults with dementia, without cognition test or wrong death time. In total, this study include 14,892 oldest old.

**CLHLS Survey form 1998-2018**

Total participants 43,487

1998 wave (9,093 newly recruited)

2000 wave (6,368 newly recruited)

2002 wave (9,748 newly recruited)

2005 wave (7,459 newly recruited)

2008 wave (9,479 newly recruited)

2011 wave (1,340 newly recruited)

9,202 participants aged 79 and younger were excluded

4,965 participants lost-to-follow-up at the first follow-up survey were exclude

29,320 oldest old met the inclusion criteria

1998 wave (8,099)

2000 wave (5,387)

2002 wave (4,262)

2005 wave (4,516)

2008 wave (6,267)

2011 wave (789)

Exclude:

Baseline 1998 to 2000: 3355 died

Baseline 2000 to 2002: 1732 died

Baseline 2002 to 2005: 2410 died

Baseline 2005 to 2008: 2574 died

Baseline 2008 to 2011: 3286 died

Baseline 2011 to 2014: 280 died

in the base 1998

Exclude:

236 dementia participants

361 participants without cognition test

194 participants with wrong death time

in the base 1998

In total, 14,892 oldest old met the inclusion criteria

10904 died and 3987 censored

**Supplementary material 2** Cohort Selection Criteria, Chinese Longitudinal Healthy Longevity Study (CLHLS), surveyed form 1998-2018.

**Supplementary material 3.** Classification of cognitive decline about ten categories.

| **Classification of cognitive decline** | **MMSE score at first cognitive function test** | **MMSE score at the second cognitive function test** | **Fluctuation range** |
| --- | --- | --- | --- |
| High normal cognitive function, maintain function (C0) | 28-30 | 28-30 | (-2, 2) |
| High normal cognitive function decline to low normal cognitive function (C1) | 28-30 | 24-27 | （1, 6） |
| high normal cognitive function decline to mild cognitive impairment (C2) | 28-30 | 18-23 | （5, 12） |
| high normal cognitive function decline to severe cognitive impairment (C3) | 28-30 | 0-17 | （11, 30） |
| Low normal cognitive function, maintain function (C4) | 24-27 | 24-30 | (-6, 3) |
| Low normal cognitive function decline to mild cognitive impairment (C5) | 24-27 | 18-23 | (1, 9) |
| Low normal cognitive function decline to severe cognitive impairment (C6) | 24-27 | 0-17 | (7, 27) |
| Mild cognitive impairment, maintain function (C7) | 18-23 | 18-30 | (-12, 5) |
| Mild cognitive impairment to decline to severe cognitive impairment (C8) | 18-23 | 0-17 | (1, 23) |
| Severe impairment, maintain function (C9) | 0-17 | 0-30 | (-30, 0) |

**Supplementary material 4.** Testing the Proportional Hazard Assumption Based on the Schoenfeld Residuals and Kaplan-Meier curves by different cognitive declined categorized by MMSE.

We tested the proportional hazard assumption by Kaplan-Meier curves when cognitive decline was classified as a categorized variable, and the linear regression of the scaled Schoenfeld residuals with time was conducted when cognitive decline was continuous variable. We found that the proportional hazards assumption was not severely violated. The Kaplan-Meier curves method does not work well for cognitive decline with many levels (eFigure1). Although Kaplan-Meier curves crossed each other, routine tests on large samples are unusually sensitive and testing the time dependent covariates with schoenfeld P = 0.08 (eFigure2). Therefore, the assumption for the proportional hazards model might have been not severely violated.


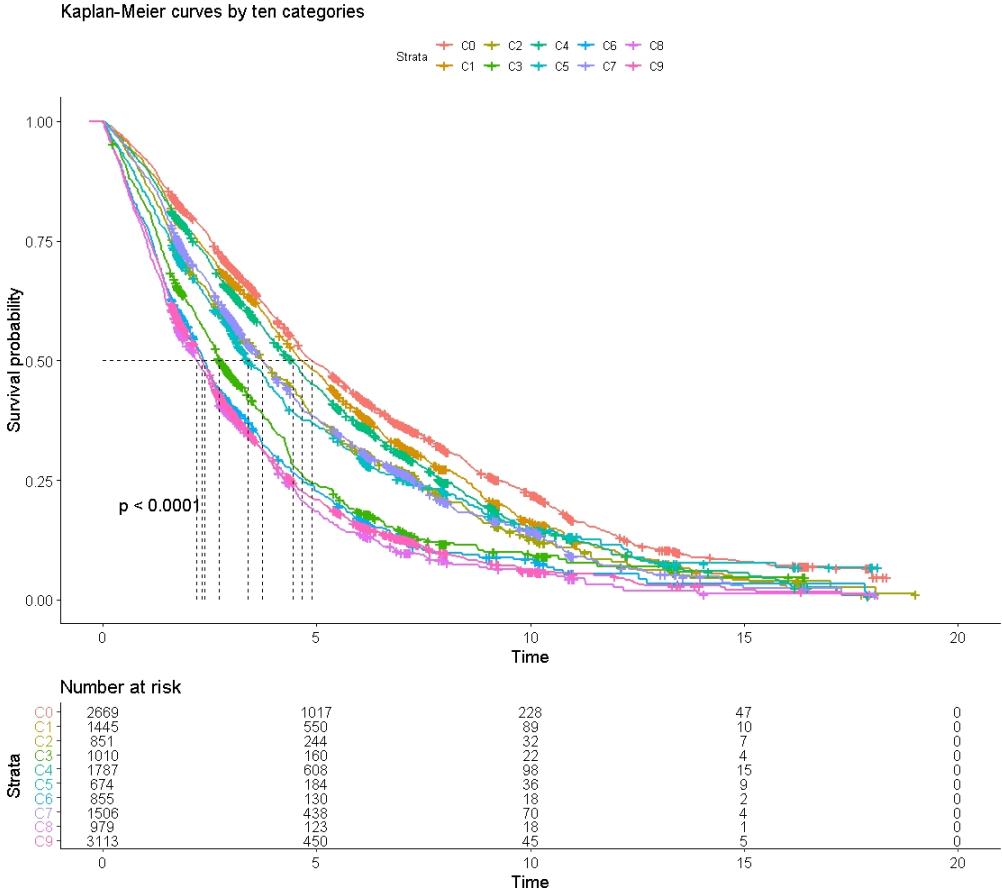


**eFigure 1.** Kaplan-Meier Curve for ten categories by the baseline MMSE score.


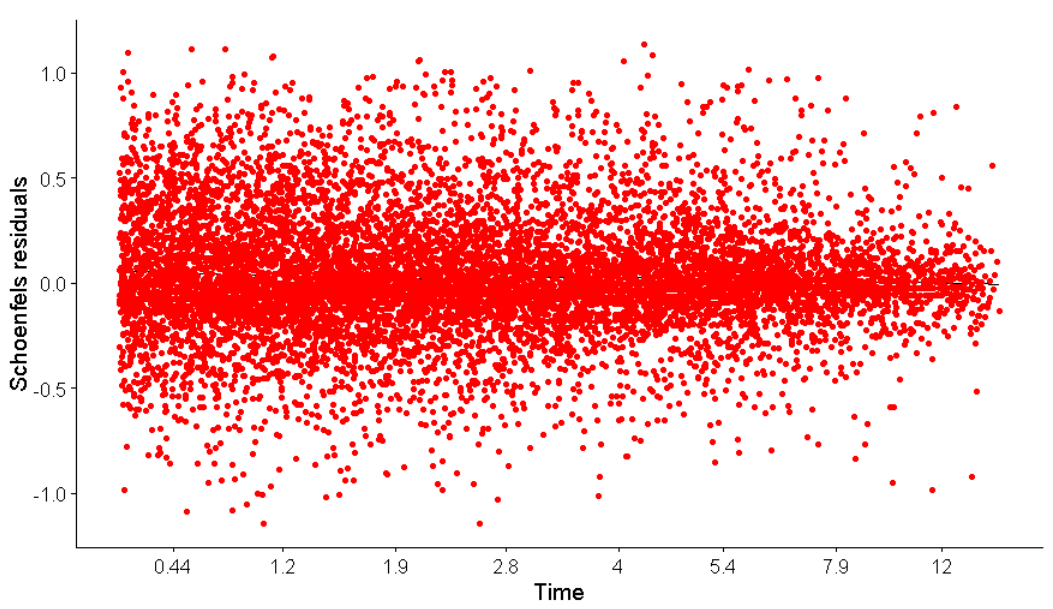


**eFigure 2. Testing the Proportional Hazard Assumption Based on the Schoenfeld Residuals.**

| **Supplementary material 5.** Baseline characteristics of the study participants of six successive and non-overlapping cohorts categorized by detailed cognitive decline. | | | | | | | | | | | | |
| --- | --- | --- | --- | --- | --- | --- | --- | --- | --- | --- | --- | --- |
| Variables | C0  (n = 2670) | C1  (n = 1445) | C2  (n = 851) | C3  (n = 1010) | C4  (n = 1787) | C5  (n = 674) | C6  (n = 855) | C7  (n = 1506) | C8  (n = 979) | C9  (n = 3114) | Total  (n = 14891) | *p* |
| Age, Mean±SD | 86.4±5.9 | 87.2±6.1 | 88.6±6.2 | 91.3±6.7 | 87.9±6.5 | 90.1±7.0 | 92.9±6.8 | 90.1±7.1 | 93.8±6.9 | 95.1±6.7 | 90.3±7.3 | < 0.001 |
| Sex, n (%) |  |  |  |  |  |  |  |  |  |  |  | < 0.001 |
| Male | 1657 (62) | 741 (51) | 392 (46) | 457 (45) | 844 (47) | 224 (33) | 275 (32) | 512 (34) | 232 (24) | 724 (23) | 6058 (41) |  |
| Female | 1013 (38) | 704 (49) | 459 (54) | 553 (55) | 943 (53) | 450 (67) | 580 (68) | 994 (66) | 747 (76) | 2390 (77) | 8833 (59) |  |
| Survival status, n (%) |  |  |  |  |  |  |  |  |  |  |  | < 0.001 |
| Censored | 920 (34) | 431 (30) | 238 (28) | 257 (25) | 547 (31) | 188 (28) | 186 (22) | 424 (28) | 179 (18) | 617 (20) | 3987 (27) |  |
| Death | 1750 (66) | 1014 (70) | 613 (72) | 753 (75) | 1240 (69) | 486 (72) | 669 (78) | 1082 (72) | 800 (82) | 2497 (80) | 10904 (73) |  |
| Educational background , n (%) |  |  |  |  |  |  |  |  |  |  |  | < 0.001 |
| Illiterate | 1165 (44) | 780 (54) | 542 (64) | 636 (63) | 1101 (62) | 494 (73) | 643 (75) | 1150 (76) | 803 (82) | 2654 (85) | 9968 (67) |  |
| Non-illiterate | 1505 (56) | 665 (46) | 309 (36) | 374 (37) | 686 (38) | 180 (27) | 212 (25) | 356 (24) | 176 (18) | 460 (15) | 4923 (33) |  |
| Current spouse status, n (%) |  |  |  |  |  |  |  |  |  |  |  | < 0.001 |
| Have spouse | 955(36) | 413(29) | 206(24) | 206(20) | 416(23) | 123(18) | 131(15) | 284(19) | 101(10) | 296(10) | 3131(21) |  |
| Have no spouse | 1715(64) | 1032(71) | 645(76) | 804(80) | 1371(77) | 551(82) | 724(85) | 1222(81) | 878(90) | 2818(90) | 11760(79) |  |
| Residence, n (%) |  |  |  |  |  |  |  |  |  |  |  | < 0.001 |
| Rural | 1330 (50) | 726 (50) | 382 (45) | 449 (44) | 776 (43) | 281 (42) | 325 (38) | 584 (39) | 374 (38) | 1064 (34) | 6291 (42) |  |
| Urban | 1340 (50) | 719 (50) | 469 (55) | 561 (56) | 1011 (57) | 393 (58) | 530 (62) | 922 (61) | 605 (62) | 2050 (66) | 8600 (58) |  |
| Living pattern, n (%) |  |  |  |  |  |  |  |  |  |  |  | < 0.001 |
| With family | 2192 (82) | 1126 (78) | 676 (79) | 825 (82) | 1440 (81) | 524 (78) | 710 (83) | 1177 (78) | 810 (83) | 2620 (84) | 12100 (81) |  |
| Without family | 478 (18) | 319 (22) | 175 (21) | 185 (18) | 347 (19) | 150 (22) | 145 (17) | 329 (22) | 169 (17) | 494 (16) | 2791 (19) |  |
| Regular exercise, n (%) |  |  |  |  |  |  |  |  |  |  |  | < 0.001 |
| No | 1513 (57) | 870 (60) | 545 (64) | 685 (68) | 1142 (64) | 459 (68) | 629 (74) | 1102 (73) | 774 (79) | 2621 (84) | 10340 (69) |  |
| Yes | 1157 (43) | 575 (40) | 306 (36) | 325 (32) | 645 (36) | 215 (32) | 226 (26) | 404 (27) | 205 (21) | 493 (16) | 4551 (31) |  |
| Current smoke status, n(%) |  |  |  |  |  |  |  |  |  |  |  | < 0.001 |
| No | 2071 (78) | 1148 (79) | 667 (78) | 830 (82) | 1446 (81) | 568 (84) | 720 (84) | 1263 (84) | 858 (88) | 2755 (88) | 12326 (83) |  |
| Yes | 599 (22) | 297 (21) | 184 (22) | 180 (18) | 341 (19) | 106 (16) | 135 (16) | 243 (16) | 121 (12) | 359 (12) | 2565 (17) |  |
| Current drink status, n (%) |  |  |  |  |  |  |  |  |  |  |  | < 0.001 |
| No | 1973 (74) | 1093 (76) | 663 (78) | 786 (78) | 1365 (76) | 552 (82) | 702 (82) | 1183 (79) | 825 (84) | 2599 (83) | 11741 (79) |  |
| Yes | 697 (26) | 352 (24) | 188 (22) | 224 (22) | 422 (24) | 122 (18) | 153 (18) | 323 (21) | 154 (16) | 515 (17) | 3150 (21) |  |
| DD, n (%) |  |  |  |  |  |  |  |  |  |  |  | < 0.001 |
| No | 1722 (64) | 948 (66) | 560 (66) | 703 (70) | 1188 (66) | 489 (73) | 599 (70) | 1102 (73) | 714 (73) | 2378 (76) | 10403 (70) |  |
| Yes | 948 (36) | 497 (34) | 291 (34) | 307 (30) | 599 (34) | 185 (27) | 256 (30) | 404 (27) | 265 (27) | 736 (24) | 4488 (30) |  |
| Disability of ADL, n (%) |  |  |  |  |  |  |  |  |  |  |  | < 0.001 |
| No | 2466 (92) | 1276 (88) | 740 (87) | 817 (81) | 1488 (83) | 546 (81) | 620 (73) | 1180 (78) | 619 (63) | 1763 (57) | 11515 (77) |  |
| Yes | 204 (8) | 169 (12) | 111 (13) | 193 (19) | 299 (17) | 128 (19) | 235 (27) | 326 (22) | 360 (37) | 1351 (43) | 3376 (23) |  |
| Housework, n (%) |  |  |  |  |  |  |  |  |  |  |  | < 0.001 |
| Often | 1247 (47) | 673 (47) | 379 (45) | 369 (37) | 721 (40) | 239 (35) | 218 (25) | 542 (36) | 231 (24) | 593 (19) | 5212 (35) |  |
| Sometimes | 560 (21) | 290 (20) | 157 (18) | 189 (19) | 346 (19) | 149 (22) | 174 (20) | 308 (20) | 184 (19) | 453 (15) | 2810 (19) |  |
| Never | 863 (32) | 482 (33) | 315 (37) | 452 (45) | 720 (40) | 286 (42) | 463 (54) | 656 (44) | 564 (58) | 2068 (66) | 6869 (46) |  |
| Gardenwork, n (%) |  |  |  |  |  |  |  |  |  |  |  | < 0.001 |
| Often | 339 (13) | 136 (9) | 75 (9) | 68 (7) | 119 (7) | 36 (5) | 32 (4) | 66 (4) | 24 (2) | 48 (2) | 943 (6) |  |
| Sometimes | 225 (8) | 95 (7) | 48 (6) | 59 (6) | 117 (7) | 28 (4) | 36 (4) | 75 (5) | 31 (3) | 71 (2) | 785 (5) |  |
| Never | 2106 (79) | 1214 (84) | 728 (86) | 883 (87) | 1551 (87) | 610 (91) | 787 (92) | 1365 (91) | 924 (94) | 2995 (96) | 13163 (88) |  |
| Read, n (%) |  |  |  |  |  |  |  |  |  |  |  | < 0.001 |
| Often | 616 (23) | 233 (16) | 92 (11) | 91 (9) | 172 (10) | 41 (6) | 37 (4) | 60 (4) | 20 (2) | 60 (2) | 1422 (10) |  |
| Sometimes | 351 (13) | 158 (11) | 77 (9) | 98 (10) | 151 (8) | 29 (4) | 41 (5) | 70 (5) | 34 (3) | 58 (2) | 1067 (7) |  |
| Never | 1703 (64) | 1054 (73) | 682 (80) | 821 (81) | 1464 (82) | 604 (90) | 777 (91) | 1376 (91) | 925 (94) | 2996 (96) | 12402 (83) |  |
| Watch tv or listen radio, n (%) |  |  |  |  |  |  |  |  |  |  |  | < 0.001 |
| Often | 1416 (53) | 680 (47) | 370 (43) | 389 (39) | 726 (41) | 224 (33) | 232 (27) | 469 (31) | 216 (22) | 541 (17) | 5263 (35) |  |
| Sometimes | 718 (27) | 398 (28) | 240 (28) | 277 (27) | 511 (29) | 193 (29) | 232 (27) | 464 (31) | 263 (27) | 648 (21) | 3944 (26) |  |
| Never | 536 (20) | 367 (25) | 241 (28) | 344 (34) | 550 (31) | 257 (38) | 391 (46) | 573 (38) | 500 (51) | 1925 (62) | 5684 (38) |  |
| Raise pet, n (%) |  |  |  |  |  |  |  |  |  |  |  | < 0.001 |
| Often | 474 (18) | 257 (18) | 154 (18) | 135 (13) | 266 (15) | 92 (14) | 97 (11) | 189 (13) | 90 (9) | 206 (7) | 1960 (13) |  |
| Sometimes | 298 (11) | 153 (11) | 105 (12) | 117 (12) | 209 (12) | 86 (13) | 106 (12) | 167 (11) | 114 (12) | 247 (8) | 1602 (11) |  |
| Never | 1898 (71) | 1035 (72) | 592 (70) | 758 (75) | 1312 (73) | 496 (74) | 652 (76) | 1150 (76) | 775 (79) | 2661 (85) | 11329 (76) |  |
| Hypertension, n (%) |  |  |  |  |  |  |  |  |  |  |  | < 0.001 |
| No | 413 (15) | 231 (16) | 135 (16) | 148 (15) | 259 (14) | 134 (20) | 121 (14) | 259 (17) | 126 (13) | 398 (13) | 2224 (15) |  |
| Yes | 2257 (85) | 1214 (84) | 716 (84) | 862 (85) | 1528 (86) | 540 (80) | 734 (86) | 1247 (83) | 853 (87) | 2716 (87) | 12667 (85) |  |
| Respiratory disease, n (%) |  |  |  |  |  |  |  |  |  |  |  | 0.097 |
| No | 2820 (91) | 2375 (89) | 1286 (89) | 774 (91) | 914 (90) | 1568 (88) | 603 (89) | 763 (89) | 1359 (90) | 874 (89) | 13336 (90) |  |
| Yes | 294 (9) | 295 (11) | 159 (11) | 77 (9) | 96 (10) | 219 (12) | 71 (11) | 92 (11) | 147 (10) | 105 (11) | 1555 (10) |  |
| Heart disease, n (%) |  |  |  |  |  |  |  |  |  |  |  | 0.018 |
| No | 2468(92) | 1334(92) | 790(93) | 951(94) | 1655(93) | 628(93) | 802(94) | 1405(93) | 919(94) | 2951(95) | 13903(93) |  |
| Yes | 202(8) | 111(8) | 61(7) | 59(6) | 132(7) | 46(7) | 53(6) | 101(7) | 60(6) | 163(5) | 988(7) |  |
| High BP, n (%) |  |  |  |  |  |  |  |  |  |  |  | < 0.001 |
| No | 1501 (56) | 877 (61) | 492 (58) | 632 (63) | 991 (55) | 366 (54) | 514 (60) | 799 (53) | 580 (59) | 1844 (59) | 8596 (58) |  |
| Yes | 1169 (44) | 568 (39) | 359 (42) | 378 (37) | 796 (45) | 308 (46) | 341 (40) | 707 (47) | 399 (41) | 1270 (41) | 6295 (42) |  |

**Note:** C0: High normal cognitive function, maintain function; C1: High normal cognitive function decline to low normal cognitive function;

C2: High normal cognitive function decline to mild cognitive impairment; C3: High normal cognitive function decline to severe cognitive impairment;

C4: Low normal cognitive function, maintain function; C5: Low normal cognitive function decline to mild cognitive impairment;

C6: Low normal cognitive function decline to severe cognitive impairment; C7: Mild cognitive impairment, maintain function;

C8:Mild cognitive impairment, maintain function; C9: Sever cognitive impairment, maintain function;

SD: standard deviation; ADL: activities of daily living; DD: dietary diversity; BP: blood pressure; IQR: interquartile range.

*The differences between groups were tested by Kruskal-Wallis test or Cochran Mantel Haenszel test

**Supplementary material 6.** Association of cognitive decline with mortality in the ten categories stratified by age.

| Add variables for sensitivity analyses | Hazard Ratio(95% CI) | |
| --- | --- | --- |
|  | Age 65-79 years old | Above 79 years old |
| Baseline high normal cognitive function |  |  |
| High normal, maintain function | 1.00 (Reference) | 1.00 (Reference) |
| High normal to low normal | 1.06(0.96,1.16) | 1.10(1.02,1.19) |
| High normal to mild impairment | 1.28(1.13,1.44) | 1.25(1.14,1.38) |
| High normal to severe impairment | 1.62(1.42,1.84) | 1.55(1.42,1.69) |
| Baseline low normal cognitive function |  |  |
| Low normal, maintain function | 1.02(0.92,1.13) | 1.06(0.99,1.15) |
| Low normal to mild impairment | 1.09(0.94,1.27) | 1.17(1.05,1.30) |
| Low normal to severe impairment | 1.36(1.16,1.6) | 1.56(1.42,1.71) |
| Baseline mild impairment cognitive function |  |  |
| Mild impairment, maintain function | 1.03(0.88,1.19) | 1.09(1.00,1.20) |
| Mild impairment to severe impairment | 1.51(1.26,1.82) | 1.62(1.47,1.80) |
| Baseline severe impairment cognitive function |  |  |
| Severe impairment | 1.16(0.88,1.53) | 1.31(1.14,1.52) |

**Supplementary material 7. Association of cognitive decline with mortality in the continuous and four categories stratified by age.**

| Model | Continuous | P for interaction |
| --- | --- | --- |
|
| Age 65-79 years old | 1.04(1.04,1.05) | <0.05 |
| Above 79 years old | 1.05(1.04,1.05) |

**Supplementary material 8. Sensitivity analyses for the association between cognitive decline and all-cause mortality after excluding the increased cognitive score**

| Sensitivity analyses | Hazard Ratio(95% CI) |
| --- | --- |
|  | Exclude the increased cognitive score |
| Baseline high normal cognitive function |  |
| High normal, maintain function | 1.00 (Reference) |
| High normal to low normal | 1.10(1.01,1.19) |
| High normal to mild impairment | 1.25(1.14,1.38) |
| High normal to severe impairment | 1.55(1.41,1.70) |
| Baseline low normal cognitive function |  |
| Low normal, maintain function | 1.07(0.94,1.20) |
| Low normal to mild impairment | 1.20(1.07,1.34) |
| Low normal to severe impairment | 1.61(1.45,1.78) |
| Baseline mild impairment cognitive function |  |
| Mild impairment, maintain function | 1.34(1.15,1.55) |
| Mild impairment to severe impairment | 1.72(1.52,1.95) |
| Baseline severe impairment cognitive function | 1.77(1.43,2.19) |

**Supplementary material 9.** Sensitivity analyses for the association between cognitive decline and all-cause mortality after excluding comorbidities.

| Sensitivity analyses | Hazard Ratio(95% CI) |
| --- | --- |
|  | Exclude comorbidities |
| Baseline high normal cognitive function |  |
| High normal, maintain function | 1.00 (Reference) |
| High normal to low normal | 1.10(1.02,1.19) |
| High normal to mild impairment | 1.26(1.15,1.38) |
| High normal to severe impairment | 1.54(1.41,1.68) |
| Baseline low normal cognitive function |  |
| Low normal, maintain function | 1.09(1.01,1.18) |
| Low normal to mild impairment | 1.19(1.08,1.32) |
| Low normal to severe impairment | 1.60(1.46,1.76) |
| Baseline mild impairment cognitive function |  |
| Mild impairment, maintain function | 1.15(1.06,1.24) |
| Mild impairment to severe impairment | 1.72(1.57,1.88) |
| Baseline severe impairment cognitive function | 1.50(1.40,1.61) |

**Supplementary material 10.** Association of cognitive decline with mortality in the ten categories after excluding the first 0.5, 1 and 1.5 year mortality of the follow-up.

| Sensitivity analyses | Hazard Ratio(95% CI) | | | |  |  |
| --- | --- | --- | --- | --- | --- | --- |
|  | Num | Exclude death in first 0.5 year | Num | Exclude death in the first year | Num | Exclude death in the first 1.5 year |
| Baseline high normal cognitive function |  |  |  |  |  |  |
| High normal, maintain function | 2577 | 1.00 (Reference) | 2461 | 1.00 (Reference) | 2295 | 1.00 (Reference) |
| High normal to low normal | 1389 | 1.11(1.02,1.20) | 1310 | 1.10(1.01,1.19) | 1212 | 1.11(1.01,1.21) |
| High normal to mild impairment | 806 | 1.25(1.13,1.38) | 741 | 1.22(1.10,1.35) | 668 | 1.20(1.08,1.34) |
| High normal to severe impairment | 923 | 1.52(1.38,1.66) | 831 | 1.50(1.36,1.65) | 712 | 1.46(1.31,1.63) |
| Baseline low normal cognitive function |  |  |  |  |  |  |
| Low normal, maintain function | 1702 | 1.06(0.98,1.15) | 1615 | 1.07(0.99,1.17) | 1487 | 1.09(1.00,1.19) |
| Low normal to mild impairment | 624 | 1.13(1.01,1.26) | 572 | 1.10(0.98,1.24) | 517 | 1.10(0.97,1.24) |
| Low normal to severe impairment | 758 | 1.50(1.35,1.66) | 671 | 1.48(1.32,1.65) | 559 | 1.41(1.25,1.60) |
| Baseline mild impairment cognitive function |  |  |  |  |  |  |
| Mild impairment, maintain function | 1434 | 1.11(1.01,1.23) | 1311 | 1.10(1.00,1.22) | 1209 | 1.12(1.01,1.26) |
| Mild impairment to severe impairment | 862 | 1.58(1.42,1.76) | 740 | 1.53(1.36,1.72) | 604 | 1.46(1.28,1.66) |
| Baseline severe impairment cognitive function | 2757 | 1.30(1.12,1.52) | 2416 | 1.27(1.08,1.50) | 2015 | 1.26(1.05,1.51) |

**Supplementary material 11.** Association of cognitive decline with mortality in the ten categories stratified by follow-up time.

| Add variables for sensitivity analyses | Hazard Ratio(95% CI) | |
| --- | --- | --- |
|  | Follow-up time (<3 years) | Follow-up time (≥3 years) |
| Baseline high normal cognitive function |  |  |
| High normal, maintain function | 1.00 (Reference) | 1.00 (Reference) |
| High normal to low normal | 1.08(0.96,1.22) | 1.12(1.01,1.24) |
| High normal to mild impairment | 1.18(1.03,1.34) | 1.14(1.00,1.31) |
| High normal to severe impairment | 1.11(0.99,1.25) | 1.42(1.23,1.64) |
| Baseline low normal cognitive function |  |  |
| Low normal, maintain function | 0.98(0.88,1.10) | 1.09(0.98,1.21) |
| Low normal to mild impairment | 1.18(1.02,1.36) | 1.06(0.90,1.24) |
| Low normal to severe impairment | 1.24(1.10,1.40) | 1.29(1.10,1.52) |
| Baseline mild impairment cognitive function |  |  |
| Mild impairment, maintain function | 1.01(0.89,1.14) | 1.11(0.97,1.28) |
| Mild impairment to severe impairment | 1.32(1.16,1.50) | 1.42(1.19,1.69) |
| Baseline severe impairment cognitive function |  |  |
| Severe impairment | 1.22(1.02,1.46) | 1.23(0.96,1.59) |
